# Metal concentrations and bioaccessibility in urban community gardens with implications for human exposure

**DOI:** 10.1007/s10653-026-03055-5

**Published:** 2026-02-16

**Authors:** Elmira Ramazanova, Manvitha Marni, Roger Wong, Leah Gable, Zezhen Pan, Zorimar Rivera-Núñez, Daniel E. Giammar

**Affiliations:** 1 Department of Energy, Environmental and Chemical Engineering, Washington University, St. Louis, MO 63130 USA; 2 Department of Public Health and Preventive Medicine, Norton College of Medicine, SUNY Upstate Medical University, Syracuse, NY USA; 3 Department of Geriatrics, Norton College of Medicine, SUNY Upstate Medical University, Syracuse, NY USA; 4 Brown School, Washington University, St. Louis, MO 63130 USA; 5 Rutgers Department of Biostatistics and Epidemiology, School of Public Health, Rutgers University, Piscataway, NJ 08854 USA; 6 Environmental and Occupational Health Sciences Institute, Rutgers University, Piscataway, NJ 08854 USA

**Keywords:** Metal, Metalloid, Lead, Community garden, Soil, Bioaccessibility

## Abstract

Urban agriculture is growing in popularity, but toxic metals and metalloids in garden soil raise concerns about human health risks associated with gardening. Gardeners might be exposed to toxic elements because they directly handle the garden soil and grow edible produce in it. This study examined community gardens in the City of St. Louis, Missouri, US and surrounding municipalities, areas with a history of soil contamination by metals, particularly Pb. To improve the current understanding of soil contamination patterns in garden soil and implications for exposure to metals/metalloids, the study (1) measured total metal/metalloid concentrations (Pb, As, Cd, Cu, Co, Ni, Mo, Ca, Mg, Fe, and Zn) in soil from twenty gardens, (2) tested in-vitro Pb bioaccessibility in soil samples, and (3) administered surveys to gardeners. Overall, our measurements suggest that Pb is a metal of concern in St. Louis community gardens. While soil in 21% of sampled plots contained Pb concentrations above recommended thresholds for gardens, Pb bioaccessibility was low (< 5.4% of the total soil concentration), suggesting that the Pb bioavailability in the case of accidental ingestion of soil particles was limited. Total metal/metalloid concentrations in soil varied spatially across plots within gardens, highlighting the importance of sampling multiple plots. Pb and As concentrations were positively correlated with garden age. Survey results revealed the common gardening habits, the type of produce grown in urban gardens, and exposure parameters. These findings contribute to improving the design of soil sampling, providing insights for exposure assessment, and informing contamination mitigation measures.

**Supplementary Information:**

The online version contains supplementary material available at 10.1007/s10653-026-03055-5.

## Introduction

Interest in urban community gardens has been increasing in the past decades and continues to grow because of their role in providing an alternative source of fresh produce, promoting health benefits, and encouraging community participation (Bieri et al., 2024; Guitart et al., 2012). Multiple studies connect participation in community gardens with improvements in public health, food security, urban ecosystems, and community cohesion (Clarke & Jenerette, 2015; Draper & Freedman, 2010; Zick et al., 2013). However, a major concern linked to urban agriculture is the presence of metals and metalloids in soil (Hou et al., 2025). Pb, As, and Cd are metals/metalloids of particular concern due to their toxicity to humans and plants (Collin et al., 2022; Mohamed et al., 2025), as well as their carcinogenic properties (IARC, 2025).

Urban surface soils typically contain high Pb, As, and Cd concentrations (Binner et al., 2023; Nezat et al., 2017). Consequently, many urban community gardens use raised beds that are filled with clean soil brought in from outside of the city. Still, metal/metalloid concentrations in garden soil can exceed the rural background levels (Mitchell et al., 2014), potentially exposing gardeners to elevated metal/metalloid levels. This concern is particularly relevant in older industrial cities like St. Louis, Missouri, US, the focus of this study and home to chemical production facilities, municipal waste incinerators, coal-fired power plants, as well as legacy smelting and metal processing activity (Kaminski & Landsberger, 2000). Missouri was one of the leading Pb producers in the US in the nineteenth and twentieth centuries, hosting an active primary smelter in the St. Louis metropolitan area through 2013 (Thorsen, 2013). Emissions from industrial facilities and the use of metal-containing products such as lead-containing paint or gasoline, and traffic could all contribute to elevated metal content in garden soil (Clark et al., 2006; McClintock, 2012; Mitchell et al., 2014). Measurement of soil concentrations is therefore needed to determine if metals/metalloids pose risks to gardeners, to evaluate exposure, and to guide safer gardening practices and public health policies.

One way to assess whether the observed metal/metalloid concentrations in soil are excessive is to compare them to threshold values set by governmental agencies. The United States Environmental Protection Agency (USEPA) does not have federal regulations for metal/metalloid concentration in garden soil specifically, but it has established soil screening values (SSVs) designed to screen sites for further investigation to evaluate if cleanup is required. These SSVs were calculated for a scenario involving accidental ingestion of soil by children and are therefore not directly applicable to community gardens. USEPA has released separate guidance on Pb levels in community gardens, recommending 100 µg/g as a “low-risk” level, which is different from the SSV for Pb (200 µg/g). Although SSVs are available for all elements of interest in this study (Pb, As, and Cd), guidance for community gardens was released for Pb only. If garden soil contains Pb concentrations above the garden-specific “low-risk” level, gardeners should consider relocation of the garden and a change in gardening practices to minimize risks (USEPA, 2014).

Although elevated levels of metals/metalloids in soil may indicate potential risk, the critical question is how their presence translates into human exposure. Gardening activities can expose individuals to metals/metalloids through inhalation, accidental ingestion of soil, and dermal contact (Kim et al., 2014, p. 201; Malone & Shakya, 2024). Consumption of garden-grown produce is another potential exposure pathway for both gardeners and consumers, but many studies reported limited exposure associated with this pathway (Clark et al., 2008; Malone & Shakya, 2024; Spliethoff et al., 2016; Watson & Margenot, 2022). Although biomonitoring has been used to evaluate human exposure to metals/metalloids (Bramwell et al., 2022; Petit et al., 2022), isolating the contribution of contact with garden soil from other sources is complicated. Accordingly, another approach to determining whether gardening poses a health concern is measuring bioavailable concentrations of metals/metalloids in soil. After entering the body through any of these exposure pathways, only a portion of the metal/metalloid concentration in soil is bioavailable, i.e., absorbed by the human digestive system into the bloodstream. While conducting animal studies is a more accurate method to measure bioavailability, validated bioaccessibility tests can be used as low-cost and faster alternatives (Bramwell et al., 2022; Li et al., 2020). Bioaccessibility testing estimates bioavailable concentrations by simulating the uptake of metals/metalloids by the human digestive system with chemical extractions (Zia et al., 2011). Bioaccessible metal/metalloid concentrations vary widely across numerous studies, ranging from nearly zero up to 80–90% of the total concentration (Entwistle et al., 2019; Kastury et al., 2024; Li et al., 2020). Soil characteristics (organic matter content, mineral phase, and soil pH) and the bioaccessibility test conditions (temperature, pH, extractant, and soil:solution ratio) can affect the reported bioaccessible concentrations (Li et al., 2020; Zia et al., 2011).

Gardening practices can influence the extent of exposure to metals/metalloids. For example, washing produce and, when applicable, peeling or removing its outer layers before consumption can help reduce gardeners’ exposure to metals/metalloids (Kessler, 2013). While washing produce does not remove metals/metalloids absorbed by plants, it significantly (though not completely) reduces adhered soil or dust particles (Augustsson et al., 2023; Clark et al., 2008). Some gardeners are aware of additional practices to decrease exposure, such as using raised beds filled with clean soil and remediating the soil (Kim et al., 2014; Wong et al., 2018). Surveying gardeners about their habits, consumption patterns, and concerns is important not only to raise awareness about safer practices but also to improve our understanding of exposure.

The overarching goal of this study was to improve understanding of metal/metalloid content and bioaccessibility in soil from community gardens. Specific objectives were to (1) examine patterns of total metal/metalloids concentrations and bioaccessible Pb concentrations in urban garden soil, (2) evaluate gardening practices, and (3) assess their implications for exposure calculations. We combined a gardener survey, soil sampling data, and measurements of bioaccessible metal concentrations. This study serves as both a case study of St. Louis community gardens and as a broader contribution to understanding how soil metal/metalloid concentrations and gardener behavior intersect to influence exposure in community gardens.

## Materials and methods

### Study area

Soil was sampled in 20 community gardens in St. Louis City and surrounding areas in St. Louis County (Fig. S1). Gardens ranged in size from 8 individual plots to over 30, with plots typically measuring 10 feet (3 m) by 3 feet (0.9 m). All plots sampled were raised beds filled with soil brought in from another location. At the time of the study, the gardens and their raised beds ranged in age from newly established to 29 years (Table S1).

### Soil sampling and preparation for analysis

Soil samples were collected from raised beds and analyzed for total metal/metalloid content (Pb, As, Cd, Cu, Co, Ni, Mo, Zn, Ca, Mg, and Fe) in June and August 2015. In each garden, four non-adjacent plots were selected for soil sampling to provide a snapshot of plot-to-plot variability, totaling 80 samples across all 20 gardens. Samples were obtained by inserting a hollow plastic barrel (inner diameter 2.6 cm) to a depth of 9.8 cm into the soil. In each plot, soil was taken from three random spots and hand-mixed to create a composite sample; the concentration of metals/metalloids in a composite sample is representative of the entire plot. Soil samples were stored in resealable plastic bags and kept in a refrigerator. These composite samples were oven-dried at 110 °C for 24 h before digestion. Six gardens (2–4, 16–17, and 20) were revisited in November 2015 to evaluate seasonal variations in element concentrations after the harvest season. Some gardeners turned over the top layers of soil after harvest to clean up raised beds and prepare the soil for the next season.

Soil pH was measured using a pH probe in a solution containing deionized water and dried soil at a 5:1 water:soil ratio (by mass). USEPA method 3050B was used to digest soil for total metal analysis (USEPA, 1996). In this method, 0.25 g of soil was digested in 10 mL of concentrated acid (HNO_3_:HCl, 4:1 by volume) at 100 °C for 4 h. After 4 h, this mixture was diluted by a factor of five with ultrapure water and filtered through a 0.2 μm poly(ether sulfone) filter (Tisch Scientific). The solution was diluted again by a factor of ten before analysis. Digestion was done in duplicate for each plot. We also digested a reference soil with a known composition (Montana II Soil/Standard Reference Material 2711a) in duplicate to test the efficiency of our digestion method. The recovery fell within the range of 83−96% for Pb, As, Cd, Ni, Co, Mo, Cu, and Zn, but was less efficient for Fe (77–78%), Ca (68–69%), and Mg (65–67%). These values are similar to or better than those reported for these elements when testing the same material using the same method (NIST, 2013).

The Urban Soil Bioaccessible Lead Test (USBLT) was designed specifically for urban community gardens to assess the bioaccessible Pb concentrations in soils by simulating gastric fluids as a glycine solution (Chaney et al., 2011). Although it does not capture the full complexity of Pb uptake by the human body, this method was chosen for its focus on community gardens and low cost. A high correlation has been demonstrated between USBLT-derived Pb bioaccessibility and Pb bioavailability in humans and rats when tested on control and remediated soils in Joplin, MO, US, supporting the validity of this test (Zia et al., 2010). Based on the USBLT protocol, a soil sample (2.5 g) was passed through a < 2 mm sieve and combined with 25 mL of 0.4 M glycine that had been adjusted to pH 2.5. The solution was agitated on a platform shaker at 100 rpm for two hours. The solution was filtered through medium porosity filter paper (Whatman), followed by a 0.2 μm poly(ether sulfone) filter (Tisch Scientific). This test was conducted at ambient laboratory temperature (22 ± 2 °C).

### Analytical measurement and statistical analysis

The filtered digested samples and USBLT samples were analyzed with inductively coupled plasma mass spectrometry (ICP-MS, Agilent 7500ce). A multi-element analytical method was used to quantify concentrations of Pb, As, Cd, Cu, Co, Ni, Mo, Zn, Ca, Mg, and Fe. Detection limits (DLs) were quantified for each element (Table S2); concentrations below the detection limits are not reported. Quality control measures included blank measurements every 10–15 samples, monitoring internal standards, and analyzing verification standard checks every 10 samples.

Statistical calculations were conducted in MATLAB 2024a. We calculated non-parametric Kendall rank correlation coefficients to find the correlation between concentrations of the analyzed elements, the age of gardens, and pH values. Coefficient of variation (CV) was used to assess variability among metal/metalloid concentrations. CV is the ratio of the standard deviation to the average multiplied by 100.

### Gardener surveys

Four to five gardeners from each community garden (n = 93) were interviewed between June and August 2015 during soil sampling. The survey questions were divided into four categories: (A) gardening practices, (B) exposure, (C) perception of community gardening, and (D) demographics (Table S3). This study primarily focuses on questions related to gardening practices and exposure. We also report demographics of gardeners to provide basic characteristics of survey participants. The part of the survey about perceptions of community gardening was published previously (Wong et al., 2018). An additional survey was administered to garden leaders (n = 20, one leader per garden) shortly before the sampling (June 2015). This garden leader survey was mainly focused on general information about gardens, such as garden age and source of soil when the garden was established (Tables S1 and S4).

## Results and discussion

### Metal/metalloid concentrations in garden soil

**Fig. 1:**
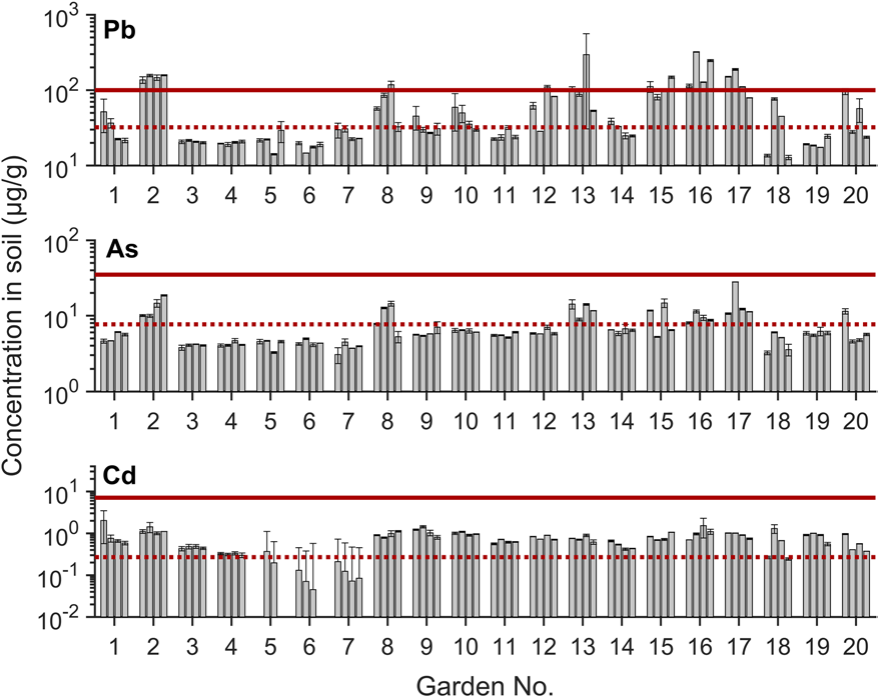
Total concentrations of (top) Pb, (middle) As, and (bottom) Cd in soil samples from urban community gardens. Each bar shows a concentration representative of an individual plot (four plots per garden, twenty gardens in total). Error bars show the minimum and maximum concentrations from duplicate digestion samples. Solid horizontal lines represent the USEPA SSVs for As and Cd in residential areas and the recommended “low-risk” level in community gardens for Pb (USEPA, 2014, 2024); dashed lines represent average Missouri surface soil concentrations, which we assumed to reflect natural baseline levels (Smith et al., 2013). Cadmium concentrations from Garden 5 (two samples) to Garden 6 (one sample) were omitted from this Figure, because they were below DL

We used SSVs for initial assessment to determine whether the metal/metalloid content in soil raises concern for gardeners, although the USEPA SSVs were not developed specifically for gardens (Table S5). Almost all of the elements analyzed in this study (Pb, As, Cd, Cu, Co, Ni, Mo, Ca, Mg, Fe, and Zn) have SSVs; the exceptions are Ca and Mg (Table S5). Although concentrations of As, Cd, Cu, Co, Ni, Mo, Fe, and Zn in soil were largely below the SSVs (Figs. 1, S2, and S3), Pb concentrations exceeded the non-carcinogenic SSV (200 µg/g) in three plots located in gardens 13 and 16 (Fig. 1). One plot from garden 5 had an outlier Cu concentration (5,389 µg/g) above the SSV (3,100 µg/g), but this was likely due to experimental error because of the wide error margin and contrast with other Cu concentrations (8–104 µg/g) (Fig. S2). In addition to SSVs, the USEPA provided a garden-specific recommendation only for one element—Pb (“low-risk” concentration, 100 µg/g) (USEPA, 2014). Seventeen plots in seven gardens (21% of all plots) had Pb concentrations above this threshold, falling into a potential risk category (Fig. 1). This finding identifies Pb as a primary metal of concern in St. Louis community gardens.

Average element concentrations in the soils of Missouri were calculated based on United States Geological Survey (USGS) data (Smith et al., 2013) (Table S5). These Missouri average values provide an estimate of natural baseline levels that primarily represent the underlying soil composition (Figs. 1, S2, and S3). Cobalt and Fe were the only elements with concentrations mostly (95–100% of all plots) at or below these baseline levels. Concentrations of Ca, Cd, Cu, and Zn were above the natural baseline concentrations in almost all plots (84%−100%). Around two-thirds of Ni, Mo, and Mg concentrations (64–73%), half of Pb concentrations (49%), and around a quarter of As concentrations (28%) in garden soils exceeded the natural baseline levels.

All soil samples in this study were obtained from raised beds. The starting soil material in raised beds is usually brought from outside the city to prevent metal/metalloids from urban surface soil from being incorporated in the garden soil. For example, a previous study conducted in Indianapolis, US, reported that Pb concentrations in clean fill soil were very low when the community garden was first established (Latimer et al., 2016). Placing barriers below raised beds is another common gardening practice to limit the fill soil’s contact with the underlying soil. Although all soil samples were collected from raised beds, our results show that plots still contained excessive Pb, As, and Cd concentrations above natural baseline levels and, in the case of Pb, above the recommended thresholds. This result could be attributed to possible recontamination by localized sources. Human activities such as traffic emissions, smelting activities, disposal of Pb-containing paint, and other industrial emissions could generate metal/metalloid-containing particles (Clark et al., 2006), which can be transported to garden soil by wind (Clark et al., 2008; Laidlaw & Filippelli, 2008). Insufficient barriers or the use of fertilizers and compost also have the potential to influence soil metal/metalloid concentrations over time. All other elements (Cu, Co, Ni, Mo, Ca, Mg, Fe, and Zn) are essential nutrients and may have been introduced to garden soils through soil amendments to promote plant growth.

The CV statistic provides insight into how variable concentrations are across all twenty gardens (Table S5). CV was lowest for Co, Fe, and Ni (18–22%). The consistency of Co, Fe, and Ni concentrations across garden plots indicates relatively uniform levels. Soil pH values were also consistent across all gardens (CV = 4%), averaging 7.33, with slightly lower pH in gardens 2 and 9 (Fig. S4). In contrast, Pb and Ca showed the highest CV (71–100%), suggesting that concentrations vary substantially garden-to-garden, possibly due to localized sources. CV values were in the range of 43–57% for all other elements (As, Cd, Cu, Mo, Zn, and Mg). Our data show a high variability for some elements not only across all twenty gardens but also across plots within a single garden (Fig. 1). For example, some plots in garden 18 have Pb concentrations 5–6 times higher than in other plots of this garden. The garden leader survey suggests that most gardens obtained soil from the same sources to fill raised beds at the time of garden establishment (Table S4). Assuming the soil had a uniform composition at establishment, the spatial variability within the garden indicates that contamination patterns differ from plot to plot. It is worth noting that each plot is managed differently because multiple gardeners share the community garden space. Gardening practices such as the addition of new soil each season, use of compost, use of raised beds and barriers, and application of fertilizers and/or pesticides can affect metal/metalloid concentrations in soil and they differ among gardeners (more details are provided in the “Gardener demographics and practices” section). Therefore, the observed plot-to-plot variability in soil concentrations could be attributed to differences in gardening practices.

Correlation analysis determines relationships between element concentrations, which may suggest possible sources (Fig. S5). We identified the following ten strongest correlations (Kendall’s tau) between elements in garden soils: Ca–Mg, Mo–Ni, Pb–Zn, Cu–Zn, Fe–Ni, Cd–Zn, Cu–Mo, As–Pb, Cd–Ni, Cu–Ni, As–Fe, Cd–Mo, Ni–Zn, Cd–Cu, Cd–Pb, and Mo–Zn (0.50–0.70, *p*-value < 6 × 10^−11^) (Fig. S5). Cobalt was an outlier in the correlation matrix because it did not correlate with any other element (− 0.27–0.14, *p*-value = 3 × 10^−4^–8 × 10^−1^). These correlations can arise from either the co-occurrence of elements in mineral phases or anthropogenic sources. To have insight into what elements tend to co-occur in parent soil material, we calculated correlation coefficients for soil concentrations in Missouri from the USGS dataset (Fig. S6) in the same way as for our garden soil dataset (Fig. S5). A strong correlation in both the USGS and garden soil datasets does not rule out the possibility of anthropogenic sources; both anthropogenic and geogenic sources can potentially result in the same correlation. However, pairs of elements with a strong correlation in the garden dataset and a weak correlation in the USGS dataset point to an anthropogenic source.

Correlations involving Pb, Cd, and As (Pb–Cd, Cd–As, As–Pb, Cd–Mo, Pb–Ca, and Pb–Zn) tend to be strong in our garden dataset (0.45–0.64, *p*-value = 6 × 10^−17^–5 × 10^−9^), but are among the weakest in the USGS baseline dataset (0.01–0.26, *p*-value = 5 × 10^−5^–9 × 10^−1^). Correlations involving Pb, Cd, As, and Zn most likely reflect the legacy of the St. Louis smelting industry (Vermillion et al., 2005). Studies of soils impacted by smelter emissions have found elevated concentrations of both Pb and Zn (Deng et al., 2016; Douay et al., 2008). Correlations between Pb, Zn, and Cd are often associated with smelting activities as well (Xing et al., 2020). Others have also connected positive Pb–Cd correlation in soil with traffic emissions and the use of phosphate fertilizers (Wuana & Okieimen, 2011).

Correlations between Mo and some elements (Mo-Cd, Mo-Ni, and Cu-Mo) also follow a similar trend; they are strong in garden soils (0.57—0.65, *p*-value = 2 × 10^–16^–9 × 10^−13^), but relatively weak in the USGS dataset (0.01–0.28, *p*-value = 8 × 10^−6^–9 × 10^−1^). The strong correlation between these elements in garden soil and their concentrations being largely above natural baseline levels suggest an anthropogenic source. Elements such as Mo, Ni, and Cu are essential for plant growth, and therefore, they are present in fertilizers and soil amendments (Wuana & Okieimen, 2011). The use of soil amendments such as compost and/or fertilizers most likely contributed to the high Mo, Zn, Ni, and Cu concentrations in raised beds. When considering the strong correlation between Mo and Cd, the source of Mo in garden soil appears to be one of the Cd sources in garden soils. It has been previously demonstrated that Cd is present as an impurity in compost and fertilizer (Wuana & Okieimen, 2011).

**Fig. 2:**
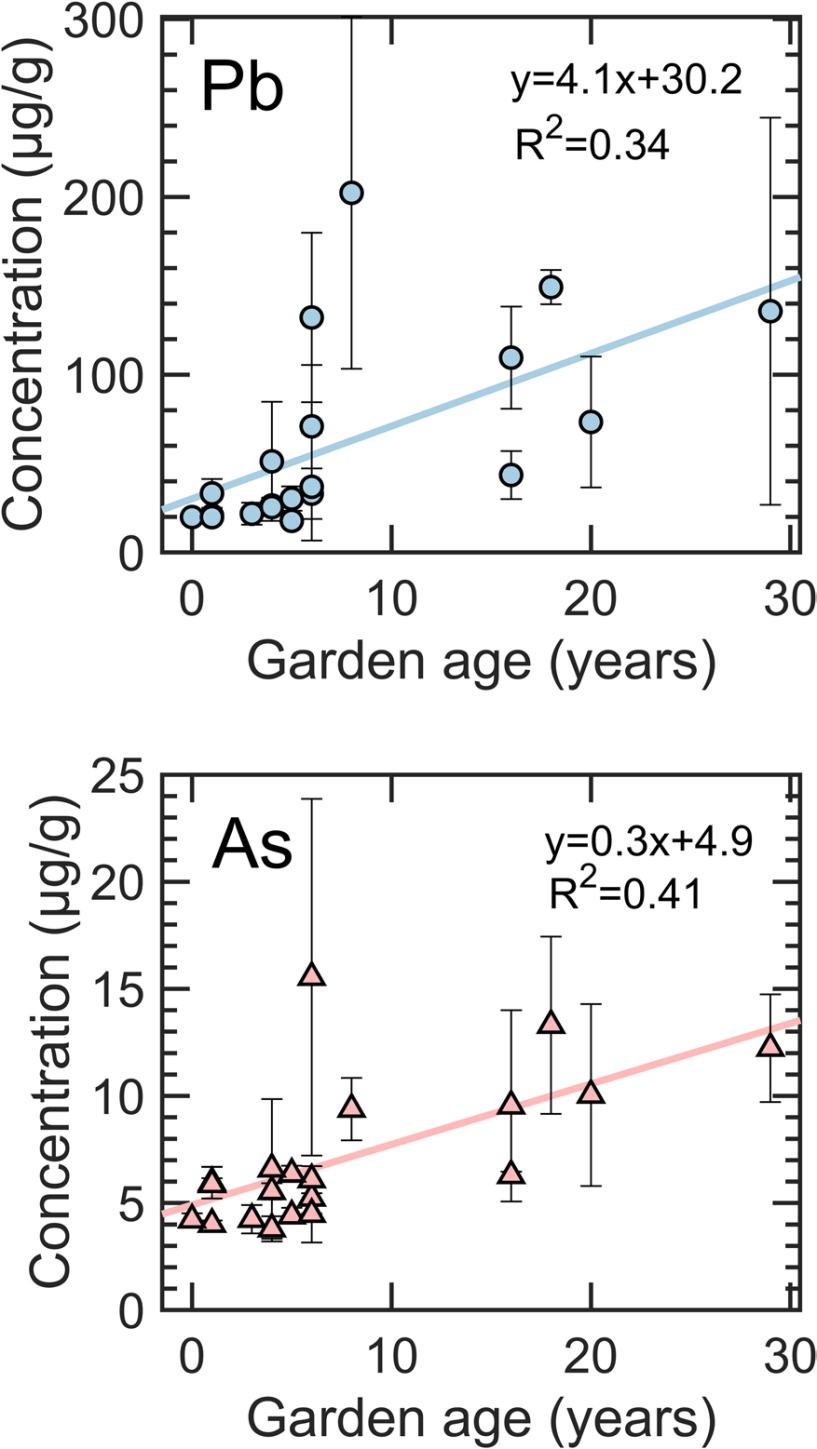
Average total (top) Pb and (bottom) As concentrations in soils as a function of garden age (as of 2015). Error bars represent the standard deviation of concentrations in four plots in each garden. The solid line represents the linear fit with the corresponding equation shown

Garden age correlates positively with some elements, most notably Pb and As (Figs. 2 and S5). Older gardens tend to have higher Pb and As concentrations than newer gardens. The linear relationship between concentrations and age is relatively weak (R^2^ = 0.3–0.4), which suggests that gardens do not consistently accumulate metal/metalloids with time. An alternative explanation for older gardens having higher Pb and As levels is that there were differences in the source of the soil used in raised beds over time. Gardens that had the most consistent and the lowest concentrations of Pb and As (gardens 3–7, 9, 11, 14, and 19) were 5 years old or less in 2015 (Fig. 1 and Table S1). Older gardens could have obtained soil from a different source with higher Pb and As concentrations or added the in-ground urban soil with high Pb and As levels to the clean fill soil at the beginning of the garden operation. It is also possible that the recently founded community gardens implemented stricter quality control for the initial fill soil compared with gardens launched 10–20 years ago.

To evaluate any seasonal differences in Pb, As, and Cd concentrations, soil in six gardens was resampled four months after the original samples were collected in July 2015. Lead concentrations were relatively consistent across four months, with the average concentration in November being just 4% higher than the average in July (Fig. S7). The average difference between fall and summer concentrations was − 3.8 µg/g (median = 0.4 µg/g). In contrast to Pb, As and Cd concentrations were higher in most plots in November than in July, with the average increase being 46% for As and 30% for Cd. The average difference between summer and fall was 1.9 µg/g (median = 3.3 µg/g) for As and 0.13 µg/g (median = 0.17 µg/g) for Cd. It is worth noting that these seasonal changes in concentration may reflect not only temporal variation, but also a variation with depth. Some beds had been turned, and produce had been harvested four months after the original sampling; these activities most likely changed the concentration profile with depth in the soil. However, overall, our results suggest that sampling time and soil turning do not influence metal/metalloid concentrations substantially.

### Bioaccessibility of lead in garden soil

**Fig. 3:**
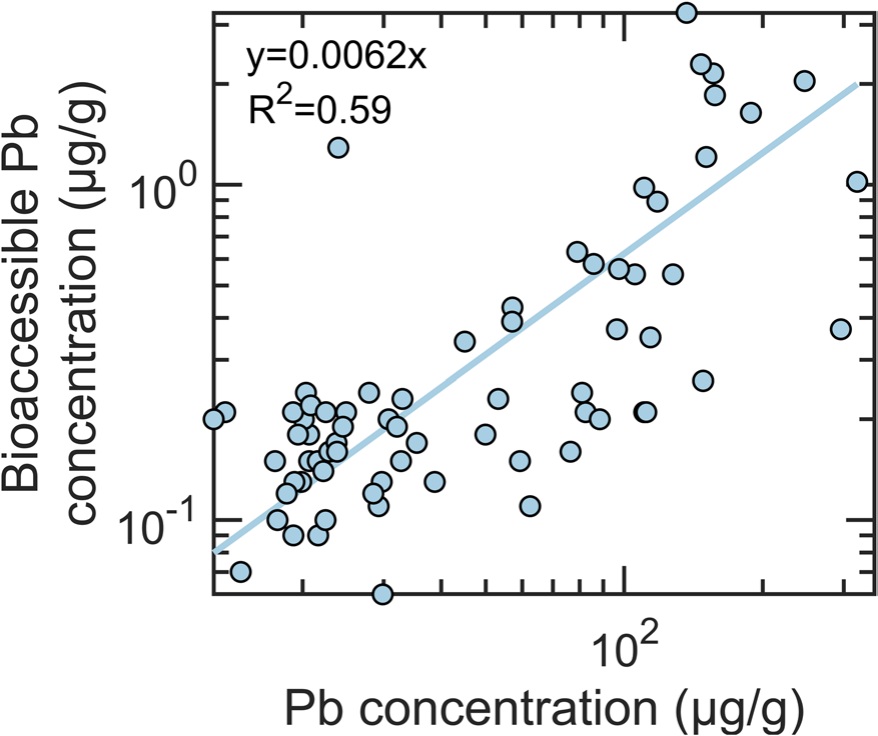
Bioaccessible Pb and total Pb concentrations in garden soils, with the solid line representing the linear fit with the corresponding equation provided in the figure

Bioaccessible Pb concentrations ranged from 0.06 to 3.26 µg/g (Fig. S8), constituting approximately 0.62% of the total Pb concentrations in soil (median = 0.66%; range = 0.1–5.4%) (Fig. 3 and Table S5). The correlation between bioaccessible and total Pb concentrations was positive (R^2^ = 0.59, Kendall’s tau = 0.51, *p*-value = 9 × 10^−10^) (Figs. 3 and S5). These low levels of bioaccessible Pb in the raised beds indicate that Pb levels in garden soils are not likely to pose a major risk for St. Louis gardeners. Even in plots with the highest soil Pb concentrations (189–320 µg/g in gardens 13, 16, and 17), bioaccessible concentrations ranged from 0.4 to 2.0 µg/g (0.3–0.9%).

Compared with other studies that used USBLT, our bioaccessibility values (< 5.4%, median = 0.66%) were low. The reported USBLT bioaccessibility values for Pb include 2–20% (median = 10%) in urban garden soil (Chaney et al., 2011; Zia et al., 2010, 2011), 23% on average in urban garden soil (Bugdalski et al., 2014), and 2–46% in contaminated soils (Beyer et al., 2016). Although the original USBLT protocol specifies a < 2 mm soil fraction, Bugdalski et al., 2014 used a < 0.25 mm fraction, which might explain the higher Pb bioaccessibility levels in their study compared with those from our study and studies by Zia et al. (Bugdalski et al., 2014; Zia et al., 2010, 2011). Although Pb in finer soil particles (< 63–150 μm) is typically more bioaccessible than in coarse particles (Saleh et al., 2025), bulk soil represents a mixture of different particle sizes. This study aimed to estimate bioaccessibility in representative soil samples for the < 2 mm soil fraction to be consistent with the USBLT protocol.

Nevertheless, our bioaccessibility values are still lower than those reported by Beyer et al. and Zia et al. at similar conditions with the same soil size fractions. It is also worth noting that the sampled soils in the current study had a relatively high pH (avg. = 7.33, median = 7.38), which is favorable for the formation of Pb-containing minerals. The highest absolute bioaccessible Pb concentration (3.26 µg/g or 2.3%) was observed in plots with the lowest soil pH values (garden 2, Figs. S4 and S8). A previous study also observed lower Pb bioavailability after treating soil with phosphorus-rich soil amendments, attributing this trend to the formation of low-solubility Pb-phosphate minerals such as pyromorphite (Pb_5_(PO_4_)_3_Cl) (Ryan et al., 2004). Other studies suggested that fertilization of garden soil might trigger precipitation of Pb-phosphate soils (Minca et al., 2013; Zia et al., 2010, 2011). Previous studies reported that urban community gardens pose low health risks to gardeners using quantitative human health risk assessment and Pb blood measurements, in agreement with our low bioaccessibility results (Bramwell et al., 2022; Lange et al., 2024).

### Gardener demographics and practices

Out of 93 gardeners surveyed, 60% were female with an average age of 50 years (median = 52, SD = 14) (Fig. S9 and Table S6). Gardeners were primarily white (65%) or black (32%). Most of the gardeners have either a bachelor’s degree (24%) or a graduate degree (41%). Although gardeners were members of their respective community gardens for an average of 4.5 years and a median of 3.8 years (SD = 3.0), gardeners had an average of 18 years and a median of 11 years (SD = 16) of gardening experience (Table S6).

The majority of the gardeners (80%) had at least one individual plot, while others shared plots with fellow gardeners (Fig. S10a). Most gardeners (91%) reported that they were using soil that was already present in the garden when they joined the community garden (Fig. S10b). At the time of the creation of the garden, most gardens (55%) obtained their soil from the same source (Table S4). Nearly all gardeners (96%) used raised beds for their plots (Fig. S10c). When asked if there was a barrier present separating the top and bottom soil layer, however, a quarter were unsure, and more than a third (37%) of all gardeners responded that they did not have a barrier present (Fig. S10c). Insufficient barriers around soil in raised beds could contribute to the observed plot-to-plot variability discussed in previous sections. Among the gardeners who used fertilizers or pesticides (29% of all 93 gardeners, n = 27), most used organic fertilizers (85%) (Figs. S10c and S10e). Around half of gardeners reported using mulching and crop rotation as soil fertility and pest control practices (Fig. S10d). The addition of compost to plots was prevalent and reported by 85% of gardeners (Fig. S10c). The compost typically contained food waste, leaves, and grass clippings and was applied once per growing season (Figs. S10f–g).

Gardeners spent an average of about 4 h (SD = 3.6) in the garden each week during the gardening season (Table S6), and 89% wore some type of protective gear (Figs. S11a and S11c). Most gardeners frequently washed their hands after gardening (90%) (Fig. S11a). Gardeners were primarily growing produce for home consumption (88%) and for charity (37%) (Fig. S11b). Most gardeners reported washing their produce before eating or cooking, primarily by rinsing it with water (Fig. S11a). The most common types of produce grown, based on the edible plant part, were fruit vegetables (89%), leaf vegetables (68%), and root/tuber vegetables (52%) (Table 1). Most gardeners (89%) consumed some produce they grew in the garden raw (Fig. S11a and Table 1). About two-thirds of all gardeners (66%) planted crops with edible parts grown underground (carrot, potato, beets, radish, turnip, onion, and garlic), and about one-third (31%) consumed them raw. These types of produce (roots, tubers, bulbs), along with herbs, tend to contain higher metal/metalloid concentrations than fruit crops (Attanayake et al., 2014; Sage et al., 2023; Spliethoff et al., 2016).

**Table 1:** Types of produce grown and eaten raw by gardeners surveyed

Produce type^a^	Examples	Grown	Eaten raw
		N (%)^b^	N (%)^c^
Fruit (culinary vegetables)	Tomato, cucumber, and zucchini	79 (85)	70 (84)
Fruit (culinary fruits)	Cantaloupe, peach, and pear	4 (4)	8 (10)
Leaf	Lettuce, spinach, and kale	63 (68)	41 (49)
Leaf (herbs)	Basil, parsley, and thyme	22 (24)	10 (12)
Roots and tubers	Carrot, potato, and turnip	48 (52)	21 (25)
Bulb and stem	Onion, garlic, and asparagus	32 (34)	19 (23)
Seed	Beans, peas, and lentils	31 (33)	24 (29)
Flower	Broccoli, cauliflower, and artichoke	11 (12)	3 (4)

^a^Produce was categorized based on the edible plant part. Fruit crops were subdivided based on commonly accepted culinary definitions, and herbs were highlighted within the leaf category

^b^Percent out of all gardeners (n = 93)

^c^Percent out of gardeners who eat raw produce (n = 83)

### Implications for environmental exposure assessment

Exposure assessment relies on soil sampling data to estimate metal/metalloid intake. While this study’s soil measurements are specific to the St. Louis area, some findings can still provide insights into the design of future community garden sampling programs. We observed variations in element concentrations across gardens in St. Louis and, in some cases, substantial plot-to-plot variations within a single garden. The plot-to-plot variability demonstrates that it will be necessary to collect multiple samples from multiple plots in a garden to get a clear picture of metal/metalloid concentrations; collection of a smaller number of samples in each garden could have obscured the ability to see plot-to-plot trends in the present study. Therefore, soil sample collection needs to balance sampling multiple plots in a single garden with sampling a few plots from many gardens. Although we observed high spatial variation in metal/metalloid levels in garden soil, sampling time appeared less significant, at least for Pb. The observed relationship between Pb and As concentrations and garden age suggests that baseline samples should be collected when gardens are first established.

In addition to soil concentrations, exposure assessment requires information about gardening practices. This study provides estimates for key exposure variables such as exposure duration (average = 18, median = 11 years) and exposure frequency (average = 4 h/week or 8.7 days/year) (Table S6). Incidental ingestion of dust/soil and consumption of produce are primary exposure pathways for gardeners (Clark et al., 2008; Spliethoff et al., 2016). A recent study found that there can be some uptake of Pb through dermal contact (Niemeier et al., 2022); our survey data suggest that dermal uptake can be assumed to be negligible since most gardeners use protective gear like gloves during gardening.

There were several limitations to this study. Some St. Louis gardens opted out of participating in this study, limiting our ability to use a random sample design. Therefore, a selection bias may be present because there could be differences between the gardens visited and those that chose not to participate. The statistical strength of the findings is also limited by the sample size. Sampling at different depths would provide insights into a concentration profile within garden soil. This research focuses on inorganic elements, but could be expanded to include organic chemicals such as pesticides, polycyclic aromatic hydrocarbons, polychlorinated biphenyls, and petroleum products. Future work should investigate solid phase speciation of Pb, mechanisms for recontamination as gardens mature, metal/metalloid concentrations in produce, mechanisms of metal/metalloid uptake by plants, and produce consumption trends to improve understanding of exposure to metals in urban community gardens.

## Conclusion

Some metals/metalloids, such as Pb, are toxic and carcinogenic elements that are present at high levels in urban surface soil due to the legacy of industrial activities. Although gardeners are aware of this and bring in soil from outside the city to fill raised beds in community gardens, the garden soil might still contain excessive concentrations of metals/metalloids. Soil sampling revealed that toxic metals/metalloids in St. Louis community garden soil were often present above Missouri’s natural baseline levels, and in the case of Pb, guidance values. These findings suggest that using clean soil in raised beds provides only partial protection. Gardeners with elevated Pb concentrations in their soil should consider exposure mitigation measures such as compost addition and/or installing barriers; raising awareness about these measures is important since the survey responses reported in this study and in the study by Wong et al. demonstrated that not all gardeners use and are aware of them (Wong et al., 2018). Pb was identified as a metal of concern. Although Pb concentrations were above recommended levels in some gardens, Pb bioaccessibility was very low (< 5.4% of total soil concentration). Although Pb does not appear to pose substantial risks in our study, gardeners should still wash their hands after gardening and wash their produce thoroughly. Learning information about garden age and soil testing is recommended for gardeners because our findings indicate that older gardens tend to have higher Pb and As concentrations. Concentrations varied substantially not only across gardens in the study area, but also across plots within some of the gardens, which highlights the need to assess spatial variation within a single garden during soil sampling efforts. Gardeners are advised to test their soil for metals/metalloids, especially in areas they or garden visitors frequent most; sampling soil at one location within the garden or plot will not provide accurate information about all plots within the garden. Our findings can help make community gardening safer by improving the design of soil sampling and by providing information about gardener exposure for risk assessment.

## Supplementary Information

Below is the link to the electronic supplementary material.

Supplementary file1 (PDF 1326 kb)

## Data Availability

Data will be made available on request.

## Declarations

### Humans and animal participants

The gardener survey was anonymous, and participation was voluntary, with verbal consent to participate obtained prior to the survey.
