# Supplementary material for: Metal concentrations and bioaccessibility in urban community gardens with implications for human exposure
Source: Environ Geochem Health. 2026 Feb 16;48(4):165. doi: 10.1007/s10653-026-03055-5 (PMC12909311; doi:10.1007/s10653-026-03055-5)
Supplement: Supplementary file 1 — Supplementary file1 (PDF 1326 kb) [file 10653_2026_3055_MOESM1_ESM.pdf]

Metal concentrations and bioaccessibility in urban community gardens with implications for human exposure

Elmira Ramazanova[1], Manvitha Marni[1], Roger Wong[2,3], Leah Gable[4], Zezhen Pan[1], Zorimar Rivera-Núñez[5,6], Daniel E. Giammar[1*]

1. Department of Energy, Environmental, and Chemical Engineering, Washington University, St. Louis, MO 63130, United States

2. Department of Public Health and Preventive Medicine, Norton College of Medicine, SUNY Upstate Medical University, Syracuse, NY, United States

3. Department of Geriatrics, Norton College of Medicine, SUNY Upstate Medical University, Syracuse, NY, United States

4. Brown School, Washington University, St. Louis, MO 63130, United States

5. Rutgers Department of Biostatistics and Epidemiology, School of Public Health, Rutgers University, Piscataway, NJ 08854 United States

6. Environmental and Occupational Health Sciences Institute, Rutgers University, Piscataway, NJ 08854

*Corresponding author: giammar@wustl.edu, 314-935-6849

Supplementary information file

Submitted to *Environmental Geochemistry and Health*

February 2026

Table of contents

Page	Contents
S3	Table S1 Garden age.
S4	Table S2 Detection limits (DLs).
S5-S6	Table S3 Survey questions.
S7	Table S4 Source of soil.<br>Table S5 Total element concentrations in garden soils.
S8	Table S6 Survey responses to open-ended numeric questions.
S9	Fig. S1 Map of the study area.
S10	Fig. S2 Total concentrations of Cu, Co, Ni, and Mo in garden soil.
S11	Fig. S3 Total concentrations of Ca, Mg, Fe, and Zn in garden soil.
S12	Fig. S4 pH values in garden soil.<br>Fig. S5 Correlation analysis for element concentrations in garden soil, pH values, and garden age.
S13	Fig. S6 Correlation analysis for element concentrations in Missouri surface soils obtained from the USGS dataset.<br>Fig. S7 Concentrations of elements in summer and fall.
S14	Fig. S8 Bioaccessible Pb concentrations by garden.<br>Fig. S9 Demographics of gardeners.
S15	Fig. S10 Survey responses about gardening practices.
S16	Fig. S11 Survey responses about exposure.<br>References

Table S1. Garden age (as of 2015).

Garden No.	Age (years)
1	6
2	18
3	1
4	0
5	3
6	5
7	4
8	20
9	1
10	16
11	4
12	6
13	29
14	5
15	16
16	8
17	6
18	6
19	1
20	4

Table S2. Detection limits (DLs) of elements in the acidified digestate (µg/L) and in soil (µg/g).

Element	DL in the digestate (µg/L)[a]	DL in soil (µg/g)[b]
Pb	0.1	0.2[c]
As	0.07	0.14
Cd	0.02	0.04
Cu	1.3	2.6
Co	0.04	0.07
Ni	0.3	0.6
Mo	0.03	0.06
Ca	10.2	20.5
Mg	8.4	16.8
Fe	11	22
Zn	1.5	3.0

[a]DLs were calculated based on the weighted calibration curve from inductively coupled plasma mass spectrometry analysis using an established procedure (Miller and Miller 2010). DL was defined as the apparent concentration of the analyte observed in the blank sample plus three standard deviations of the blank signal.

[b]We converted DLs from µg/L to µg/g to facilitate direct comparison of DLs with soil concentrations reported in Table S5 and Figs. 1, S2, S3, and S8. We converted units by multiplying DLs in the digestate by a factor of 2 since we added 0.25 g soil to 50 mL of the digestion solution and diluted it by a factor of 10.

[c]For bioaccessible Pb concentration, we added 2.5 g soil to 25 mL of the solution and diluted 10 times. Therefore, to convert DL of bioaccessible Pb concentrations, we multiply DL in the digestate by 0.1.

Table S3. Survey questions (number of gardeners interviewed = 93).

Part[a]	No.	Question	Question type	Response options
A	1	How many plots do you have at this garden? If you don't have your own plot and share plot with someone else, write "none".	Open-ended (numeric)	
A	2	Are the plots filled with soil at the beginning of the season or do you bring your own soil?	Multiple choice (select all that apply)	Filled; Own; Don't know; Other
A	3	Do you use raised beds?	Multiple choice (single response)	Yes; No
A	4	Is there a barrier added below the top of the soil?	Multiple choice (single response)	Yes; No; Don't know
A	5	Do you use any type of fertilizer or pesticide for your garden?	Multiple choice (single response)	Yes; No
A	6	If you answered Yes in Question 5, what type of fertilizer do you use?	Multiple choice (select all that apply)	Conventional (commercial); Organic (commercial); Other
A	7	Do you add compost to your garden?	Multiple choice (single response)	Yes; No
A	8	If you answered Yes in Question 7, what do you include in your compost?	Multiple choice (select all that apply)	Food waste; Manure; Leaves; Grass; Paper; Worms; Other; Don't know
A	9	If you answered Yes in Question 7, how frequently do you add compost to your garden during the season?	Multiple choice (single response)	Once; Twice; Three times; Four times; More that four times
A	10	Do you rely on any of the following practices for soil fertility and/or pest suppression?	Multiple choice (select all that apply)	Mulching; Cover crops; Nutrient management; Crop rotation
B	11	How many hours do you spend at the garden on average per week?	Open-ended (numeric)	
B	12	Do you usually wash your hands before you start gardening?	Multiple choice (single response)	Yes; No
B	13	Do you usually wash your hands after you finish gardening?	Multiple choice (single response)	Yes; No
B	14	Do you use protective gardening gear?	Multiple choice (single response)	Yes; No

B	15	If you answered Yes in Question 14, what protective gardening gear do you use?	Multiple choice (select all that apply)	Gloves; Apron/Vest; Hat; Special shirts or pants; Tick gaiters; Knee pads; Other
B	16	Do you wash your produce at home before eating or cooking?	Multiple choice (single response)	Yes; No
B	17	Do you wash your produce with anything besides water?	Multiple choice (single response)	Yes; No
B	18	What do you grow produce for?	Multiple choice (select all that apply)	Home consumption; Farmer's market/sell; Soup kitchens; Charity; Other
B	19	What produce are you growing this summer?	Open-ended (text)	
B	20	Do you eat your produce raw?	Multiple choice (single response)	Yes; No
B	21	If you answered Yes in Question 20, which produce do you eat raw?	Open-ended (text)	
D	22	For how long have you been using this garden?	Open-ended (numeric)	
D	23	For how many years have you been gardening?	Open-ended (numeric)	
D	24	What is your gender?	Open-ended (text)	
D	25	What is your age?	Open-ended (numeric)	
D	26	Race/Ethnicity	Open-ended (text)	
D	27	Highest level of education	Multiple choice (single response)	High school; Technical degree; Some college; Associate degree; Bachelor's degree; Graduate degree
D	28	What is your household income?	Multiple choice (single response)	<$20,000; $20,000-40,000; $40,000-60,000; $60,000-80,000; $80,000-100,000; >$100,000
D	29	What is your occupation?	Open-ended (text)	

[a]Survey parts: (A) gardening practices and activities, (B) exposure, (C) perception of community gardening, and (D) demographics. Part C was published in another study (Wong et al. 2018).

Table S4. Source of soil reported by garden leaders (n=20).[a]

Source name	Frequency (percentage)
Gateway Greening[b]	7 (35%)
STL composting[b]	13 (65%)
STL city	2 (10%)
Unknown	2 (10%)
Other	5 (25%)

[a]Open-ended text-based question.

[b]All garden leaders, who knew the source of soil (n=18) reported using at least one of these sources. The number of gardens that obtained soil exclusively from these sources was 11 (55%).

Table S5. Total element concentrations in garden soil.

Element	Unit	Max.	Min.	Avg.	Median	SD[a]	CV[a] (%)	Recommended levels[b]	Baseline levels[c]
Pb	µg/g	320.8	12.8	62.7	31.5	62.8	100	100	32.2
As	µg/g	28.0	3.1	7.2	5.8	4.1	57	35	7.7
Cd	µg/g	2.02	DL[d]	0.72	0.72	0.38	52	7.1	0.27
Cu	µg/g	5,389[e]	8.2	104.2	36.3	598.5	574	3,100	15.2
Co	µg/g	11.8	3.3	6.2	6.1	1.1	18	23	12.6
Ni	µg/g	23.3	6.1	15.5	15.2	3.5	22	820	14.6
Mo	µg/g	6.5	DL[d]	1.5	1.3	0.8	55	390	1.1
Ca	mg/g	97.3	6.4	28.7	20.8	20.5	71		4.3
Mg	mg/g	8.9	1.5	4.5	4.4	1.9	43		2.9
Fe	mg/g	25.2	4.8	12.3	11.9	3.6	29	55	19.1
Zn	µg/g	1,042	44.7	146.2	127.7	122.4	84	23,000	54.6
Bio-Pb[f]	µg/g	3.26	0.06	0.45	0.21	0.62	136		
	%	5.39	0.13	0.74	0.66	0.69			

[a]SD (standard deviation) and CV (coefficient of variation). CV is the only column that has a unit (%) other than µg/g or mg/g as indicated in the second column.

[b]SSV (non-carcinogenic soil screening value for children) was used for all elements (USEPA 2024), except Pb. For Pb, the recommended low-risk value for garden soil (100 µg/g) is listed instead of SSV (200 µg/g).

[c]Average element concentrations in Missouri surface soil obtained from the USGS dataset (Smith et al. 2013).

[d]Below detection limit (DL). The lowest detectable concentrations are 0.05 µg/g for Cd and 0.6 µg/g for Mo.

[e]This value is most likely an outlier; the next highest Cu concentration was 104.0 µg/g.

[f]Bio-Pb is the bioaccessible Pb concentration. We present it as absolute value (µg/g), and as % of the total Pb concentration.

Table S6. Survey responses to open-ended numerical questions (n=93).[a]

Question	Avg.	Median	SD	Min.	Max
How many hours do you spend at the garden on average per week? (Part B, Question 11)	4.0	3.0	3.6	1	24
For how long have you been using this garden? (Part D, Question 22)	4.5	3.8	3.0	1	18
For how many years have you been gardening? (Part D, Question 23)	18	11	16	1	NA[a]
What is your age? (Part D, Question 25)	50.1	52.0	13.8	24	83

[a]The maximum value was missing from the records.

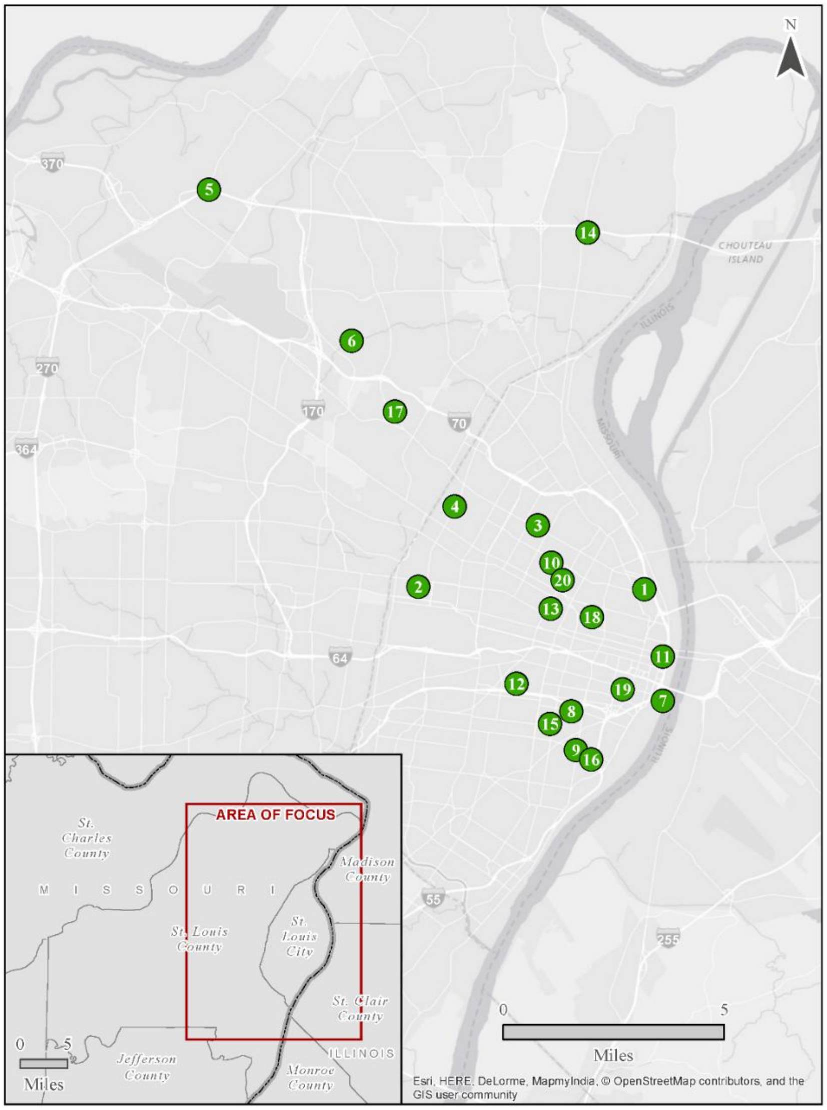

Fig. S1 Map of the study area and community gardens chosen for sampling. The bottom-left insert displays the area of focus of study.

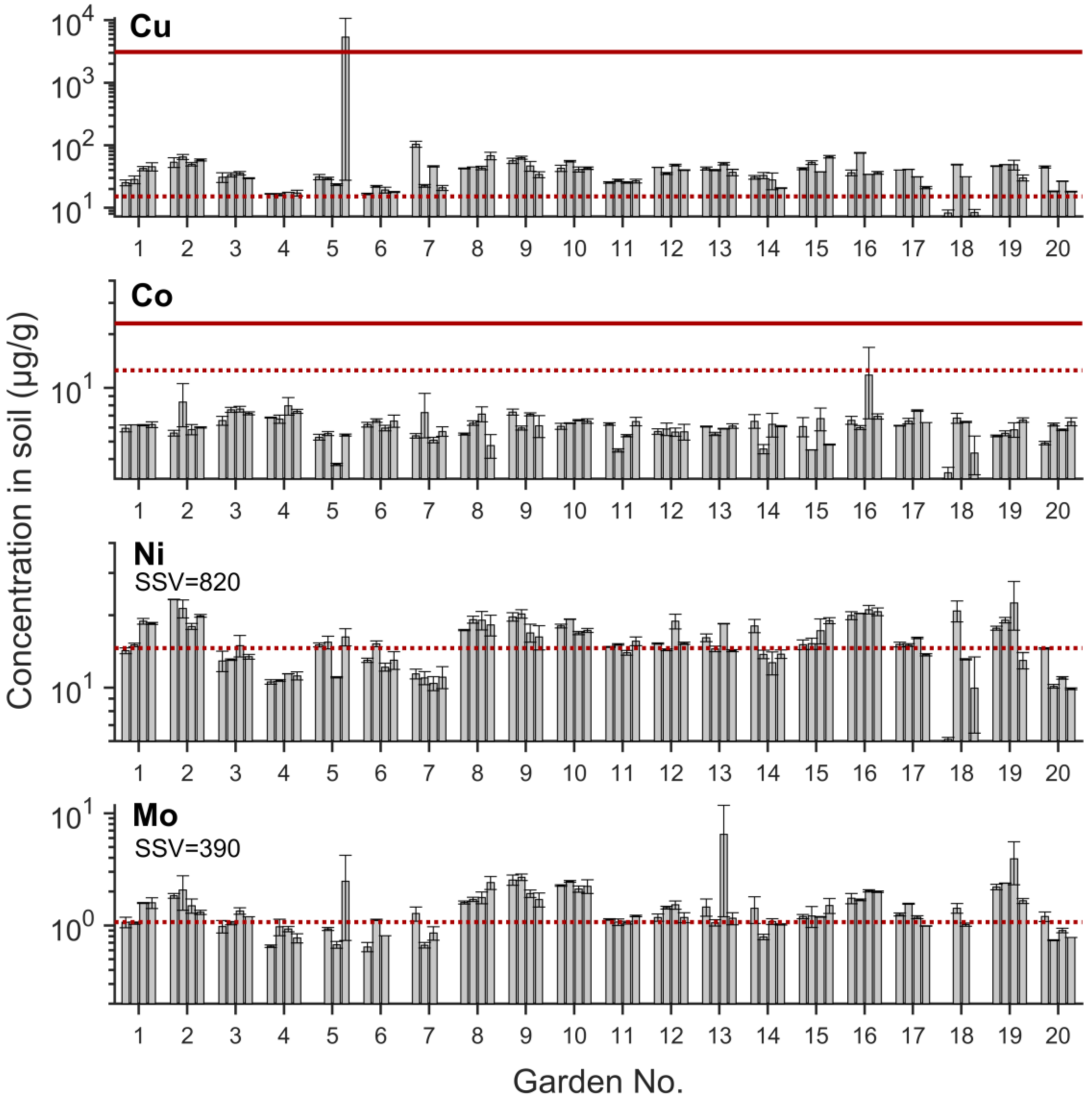

Fig. S2 Total concentrations of (top) Pb, (middle) As, and (bottom) Cd in soil samples from urban community gardens. Each bar shows a concentration representative of an individual plot (four plots per garden, twenty gardens in total). Error bars show the minimum and maximum concentrations from duplicate digestion samples. Solid lines represent EPA SSVs (USEPA 2024); the solid lines are absent in Ni and Mo panels because concentrations were lower than SSVs. Dashed lines represent average Missouri surface soil concentrations (Smith et al. 2013), which we assumed to reflect natural baseline levels. Mo concentrations from Gardens 5, 6, 7 (one sample) and Garden 18 (two samples) are omitted in the figure because they were below detection limits.

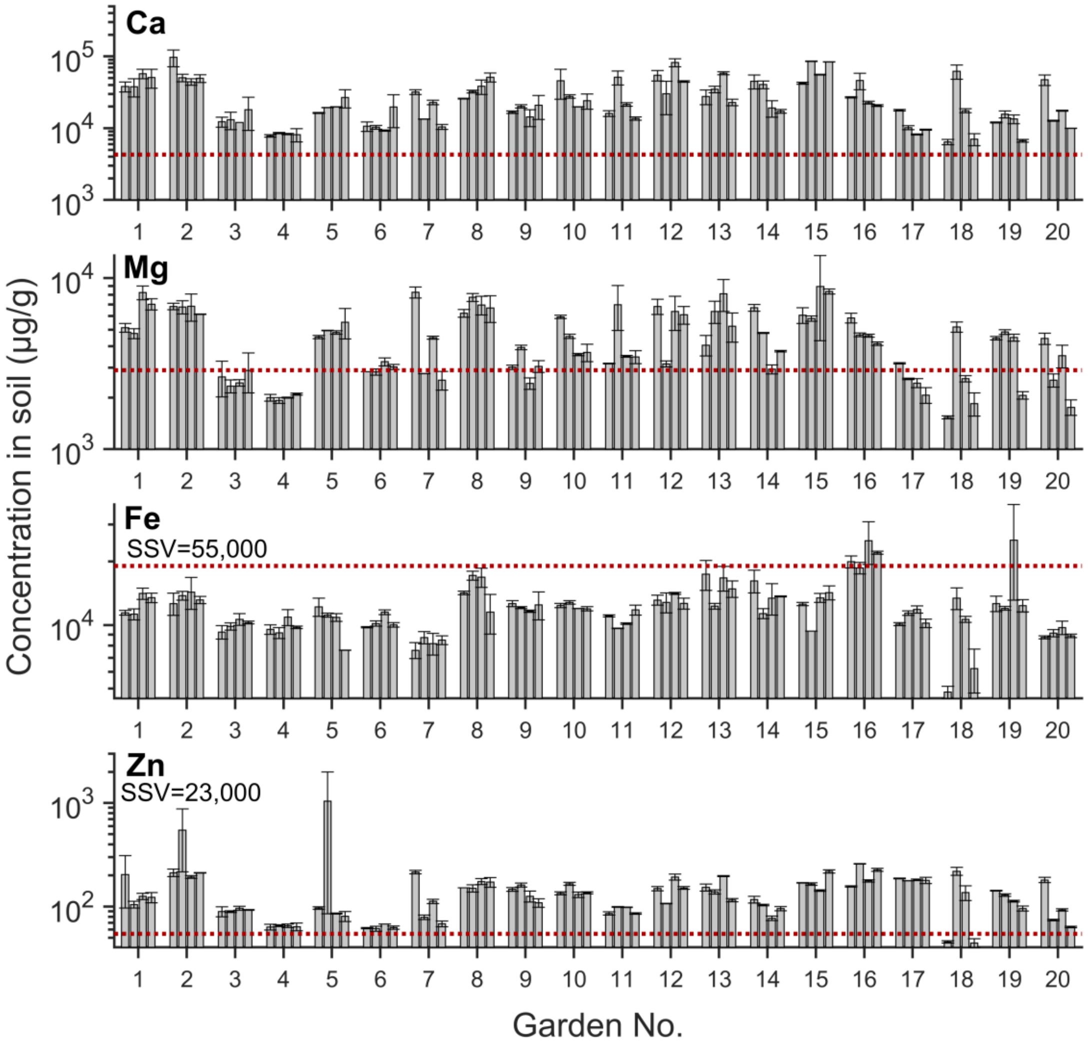

Fig. S3 Total concentrations of Ca, Mg, Fe, and Zn in soil samples from urban community gardens. Each bar shows a concentration representative of an individual plot (four plots per garden, twenty gardens in total). Error bars show the minimum and maximum concentrations from duplicate digestion samples. Ca and Mg do not have SSVs, and Fe and Zn concentrations were below SSVs. Dashed lines represent average Missouri surface soil concentrations (Smith et al. 2013), which we assumed to reflect natural baseline levels.

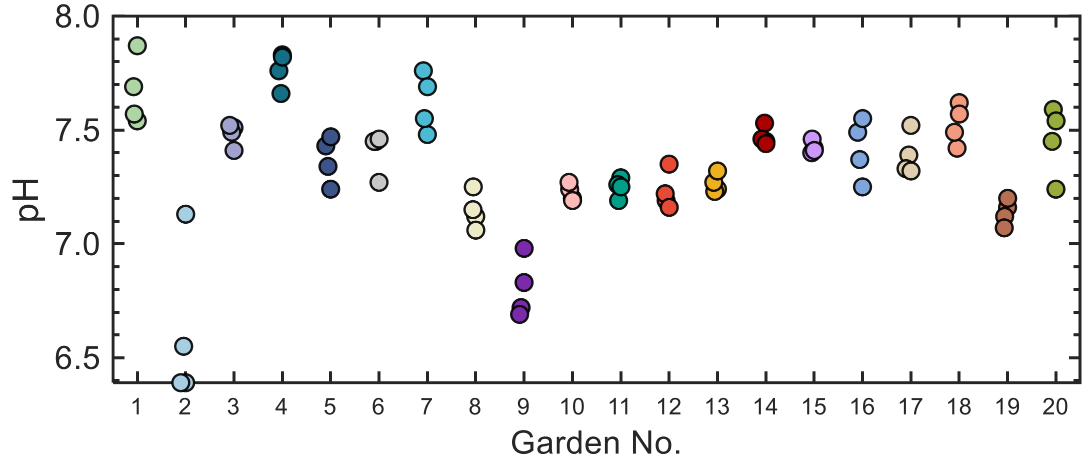

Fig. S4 pH values of garden soils.

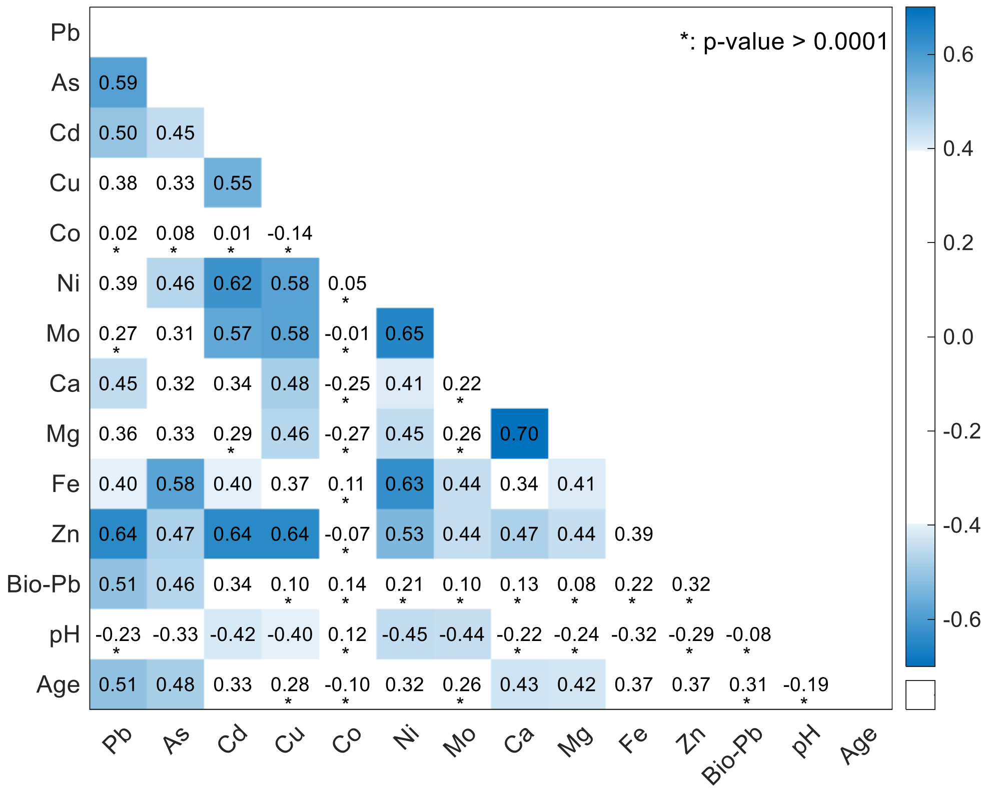

Fig. S5 Kendall rank correlation coefficients (Kendall's tau) for element concentrations in garden soil (n=80), bioaccessible Pb concentration (bio-Pb), pH values, and age of gardens. Values highlighted with an asterisk have p-values > 1×10^{-4}; all other values have p-values ≤ 1×10^{-4}.

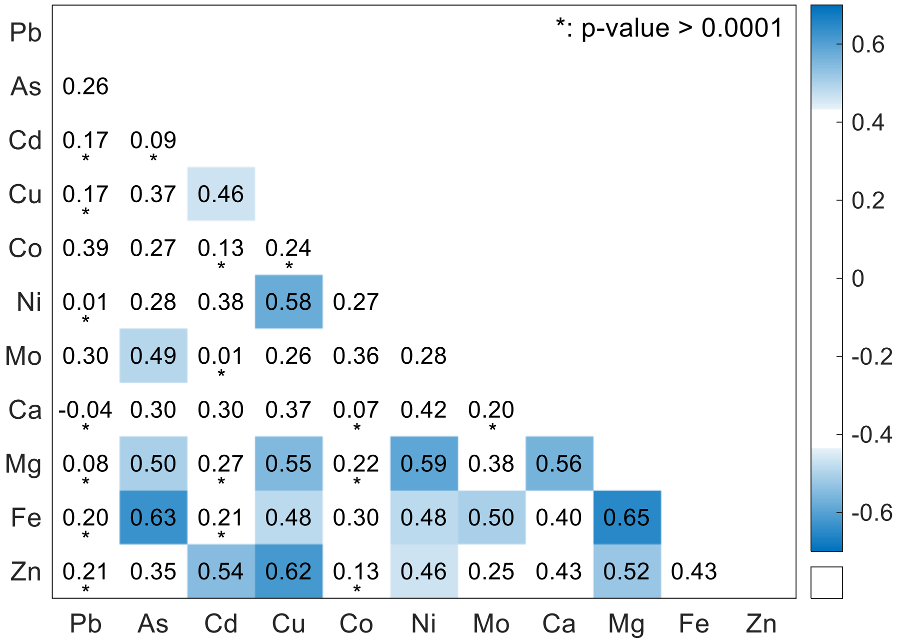

Fig. S6 Kendall rank correlation coefficients (Kendall's tau) for element concentrations in Missouri surface soil obtained from the USGS dataset (n=115) (Smith et al. 2013). Values highlighted with an asterisk have p-values > 1×10^{-4}; all other values have p-values $\leq 1\times10^{-4}$.

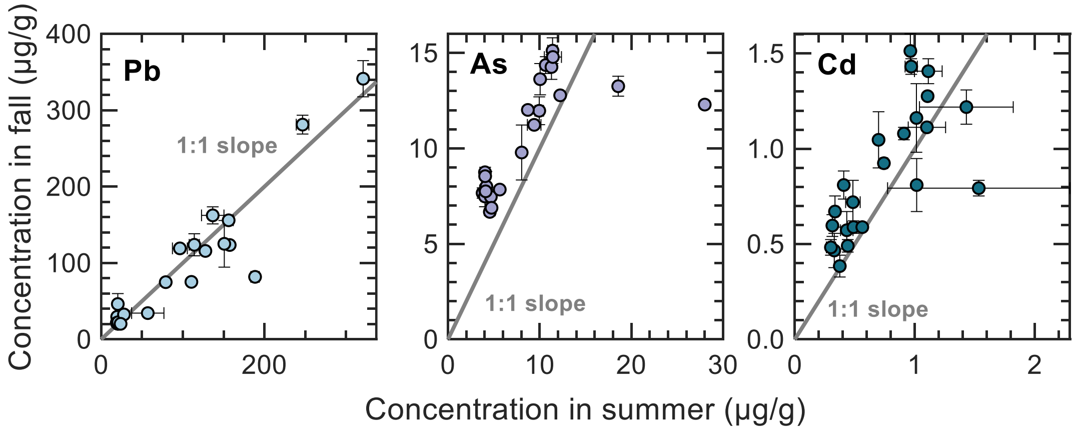

Fig. S7 Concentration of (left) Pb, (center) As, and (right) Cd in summer and fall after the produce had been harvested. Solid grey line represents 1:1 slope; markers above the line indicate that concentrations are higher in the fall than they were in the summer and vice versa.

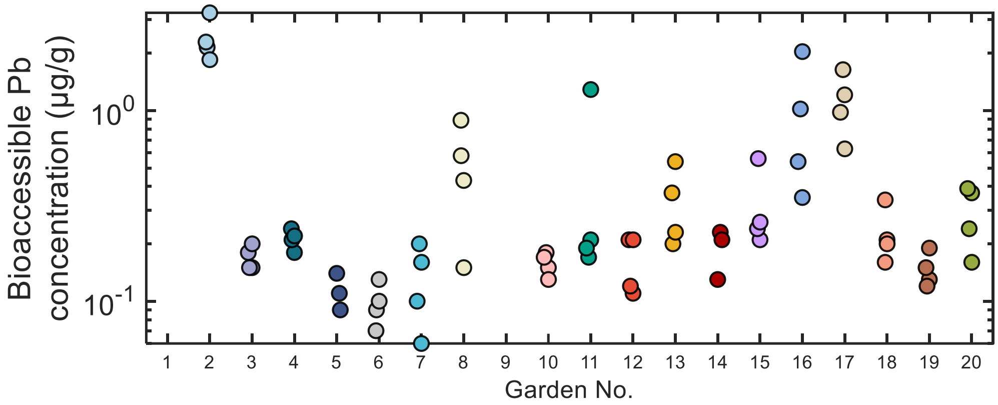

Fig. S8 Bioaccessible Pb concentrations in garden soils.

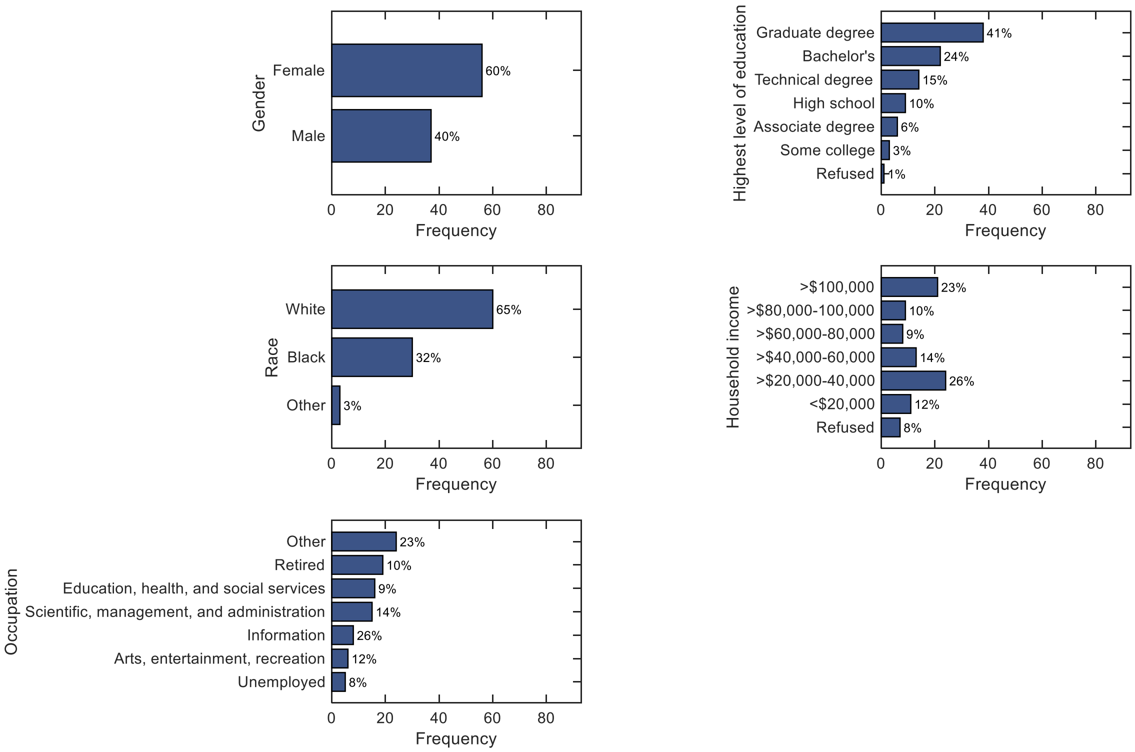

Fig. S9 Demographics of community gardeners surveyed. Bar labels show percentages (n = 93). Full question text is provided in Table S3 (Part D, Questions 24 and 26-29).

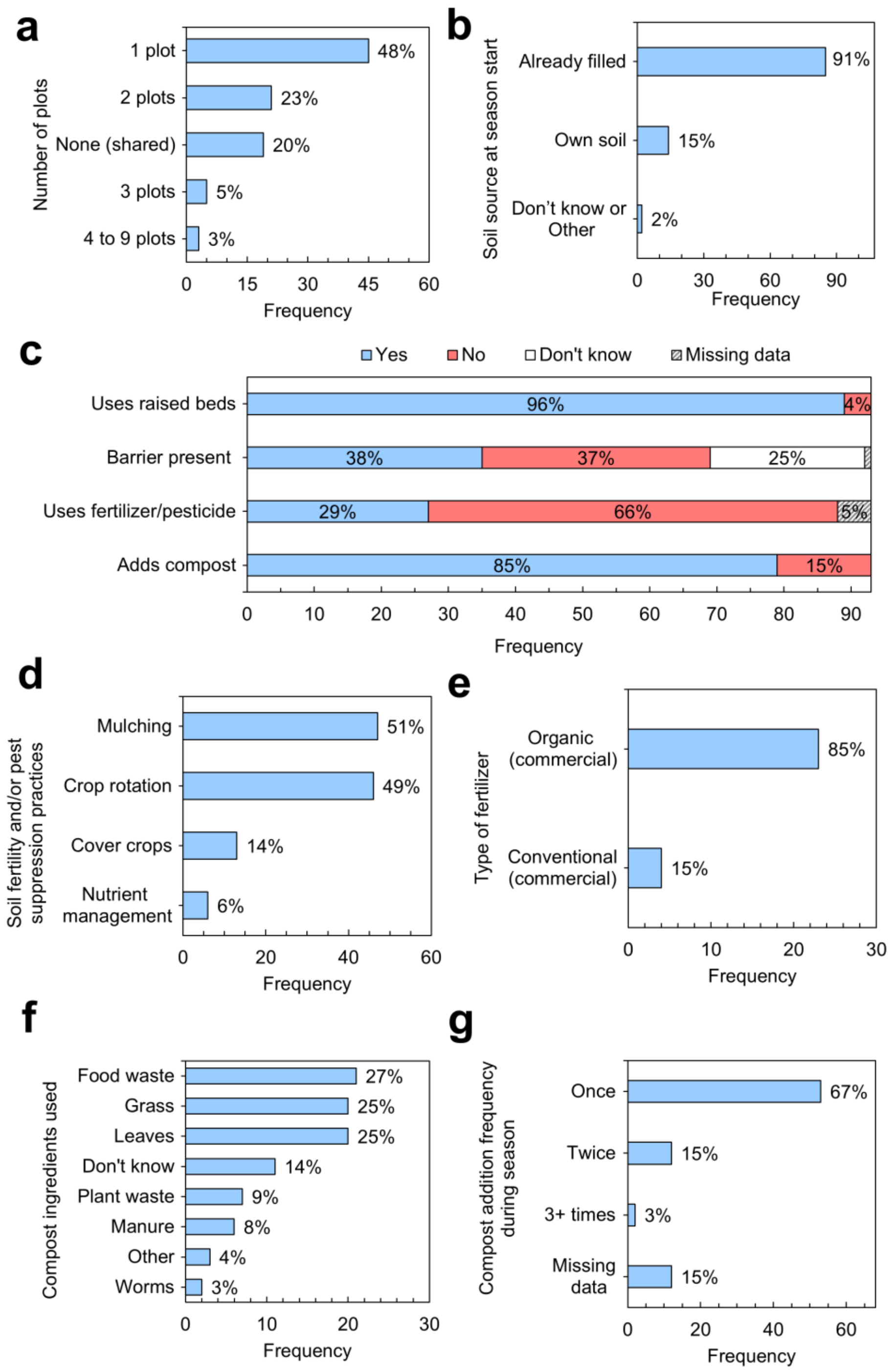

Fig. 10 Survey responses about gardening practices and activities. The total number of gardeners interviewed was 93. Bar labels show percentages; percentages are based on the total number of gardeners (n=93) in panels (a-d), the total number of gardeners who use fertilizers (n=27) in panel (e), and total number of gardeners who use compost (n=79) in panels (f-g). Full question text is provided in Table S3 (Part A, Questions 1-10). Panels (a), (c), and (g) represent single-response questions (percentages sum to 100%), whereas panels (b) and (d-f) represent “select all that apply” questions (percentages do not sum to 100%).

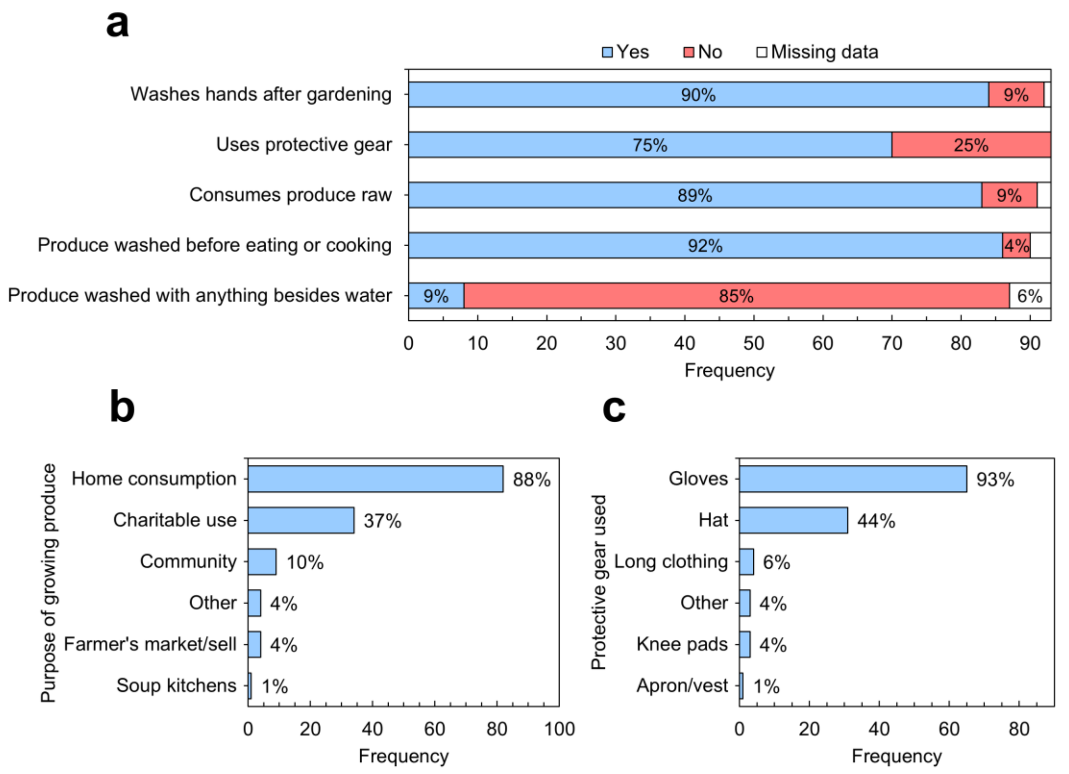

Fig. 11 Survey responses about exposure to metals/metalloids. The total number of gardeners interviewed was 93. Bar labels show percentages; percentages are based on the total number of gardeners (n=93) in panels (a) and (b) and the total number of gardeners who wear protective gear (n=70) in panel (c). Full question text is provided in Table S3 (Part B, Questions 13-18 and 20). Panels (a) represent a single-response question (percentages sum to 100%), whereas panels (b-c) represent "select all that apply" questions (percentages do not sum to 100%).

References

Smith, D. B., Cannon, W. F., Woodruff, L. G., Solano, F., Kilburn, J. E., & Fey, D. L. (2013, October 25). Geochemical and mineralogical data for soils of the conterminous United States: U.S. Geological Survey Data Series 801. https://pubs.usgs.gov/ds/801/. Accessed 26 July 2025

USEPA. (2024). Regional Screening Levels. https://www.epa.gov/risk/regional-screening-levels-rsls-generic-tables

Wong, R., Gable, L., & Rivera-Núñez, Z. (2018). Perceived Benefits of Participation and Risks of Soil Contamination in St. Louis Urban Community Gardens. *Journal of Community Health*, *43*(3), 604–610. https://doi.org/10.1007/s10900-017-0459-8
